# Dual-ATME: Dual-Branch Attention Network for Micro-Expression Recognition

**DOI:** 10.3390/e25030460

**Published:** 2023-03-06

**Authors:** Haoliang Zhou, Shucheng Huang, Jingting Li, Su-Jing Wang

**Affiliations:** 1 School of Computer, Jiangsu University of Science and Technology, Zhenjiang 212100, China; haoliangzhou6@gmail.com; 2 Key Laboratory of Behavior Sciences, Institute of Psychology, Chinese Academy of Sciences, Beijing 100101, China; wangsujing@psych.ac.cn; 3 Department of Psychology, University of the Chinese Academy of Sciences, Beijing 100049, China

**Keywords:** micro-expression recognition, attention mechanism, regions of interest

## Abstract

Micro-expression recognition (MER) is challenging due to the difficulty of capturing the instantaneous and subtle motion changes of micro-expressions (MEs). Early works based on hand-crafted features extracted from prior knowledge showed some promising results, but have recently been replaced by deep learning methods based on the attention mechanism. However, with limited ME sample sizes, features extracted by these methods lack discriminative ME representations, in yet-to-be improved MER performance. This paper proposes the Dual-branch Attention Network (Dual-ATME) for MER to address the problem of ineffective single-scale features representing MEs. Specifically, Dual-ATME consists of two components: Hand-crafted Attention Region Selection (HARS) and Automated Attention Region Selection (AARS). HARS uses prior knowledge to manually extract features from regions of interest (ROIs). Meanwhile, AARS is based on attention mechanisms and extracts hidden information from data automatically. Finally, through similarity comparison and feature fusion, the dual-scale features could be used to learn ME representations effectively. Experiments on spontaneous ME datasets (including CASME II, SAMM, SMIC) and their composite dataset, MEGC2019-CD, showed that Dual-ATME achieves better, or more competitive, performance than the state-of-the-art MER methods.

## 1. Introduction

As an important nonverbal cue for emotional understanding, facial micro-expressions (MEs) are very brief and involuntary facial expressions that usually last from 0.04 to 0.2 s [1]. In contrast to ordinary expressions, MEs are considered the unconscious leakage of genuine feelings when people try to hide them. Therefore, MEs can help reveal hidden emotions and have promising applications in many fields, such as clinical diagnosis [2] and national security [3].

Micro-expression recognition (MER) has attracted more and more attention from researchers in recent decades as a critical way of understanding human emotions [4]. In addition to traditional human-based psychological studies, many researchers have tried to investigate automatic MER methods based on computer vision techniques. In recent years, research on MER has gradually progressed with the development of published ME datasets [5,6,7,8,9,10,11,12]. There are two main approaches: methods based on Hand-crafted features [13,14,15,16,17,18] and methods based on Deep learning [19,20,21,22,23,24,25]. However, a crucial research issue for these two methods is extracting salient and discriminative features from MEs. Firstly, the subtle and fleeting properties of MEs require improvement in the representativeness of both manual features and deep learning-based features. Secondly, while deep learning has demonstrated strong capabilities of feature learning, it relies on extensive data for training. The number of spontaneous annotated ME samples is limited to only just over 2000, which limits the ability of deep learning networks to extract high-level features related to MEs. Thus, extracting discriminative features of MEs remains an area of ongoing exploration.

Enhancing MER performance by expanding the data scale is highly challenging. The data collection and annotation for ME is especially complicated. With limited data, single-scale features may not be sufficient for discriminating different categories of MEs. Combining multiple channels and effectively integrating various scale features, it is possible to improve the model’s ability to learn ME features.

In order to learn ME characteristics efficiently, we propose a simple and effective method called **Dual** -branch **AT**tention network for **M**icro-**E**xpression recognition (**Dual-ATME**), which uses feature selection, based on both Hand-crafted Attention Region Selection (**HARS**) and Automated Attention Region Selection (**AARS**), for MER. Our proposed method includes three sub-modules: Data preprocessing, Dual-ATME module and Feature fusion for ME classification.

In data preprocessing, to better capture the ME motion information, we computed dual-scale optical flow features between the onset and apex frames. Precisely, one scale feature is from the full face and the other is from the HARS-based puzzled counterpart. In the Dual-ATME module phase, in order to simultaneously learn global and local ME features, we utilize the bi-Inception network for HARS-based and AARS-based (full-face) feature extraction, respectively. Furthermore, in order to be able to better focus on ME-related features in the full-face learning network, we employ a combined channel and spatial attention mechanism module. After obtaining the features at both scales, we narrow the distance between them by means of similarity comparison, which empowers the network to refine the ME features common to both. Finally, emotional category prediction is performed by fusing the HARS-based and AARS-based features.

The main contributions of our work are as follows:

- We propose the Dual-ATME framework, which extracts HARS- and AARS-based features to perform MER. By adding a parallel artificially-selected ROI ME feature learning module to a standalone deep attention mechanism, we enable the proposed Dual-ATME to effectively learn more discriminative ME features. In particular, based on experimental results, we find that manual feature extraction, based on prior knowledge, is essential for MER with limited data size.
- We design a simple and effective joint loss to optimize feature discrimination in our proposed framework. In particular, in addition to the traditional loss for ME classification, we use a similarity comparison loss to close the distance of the dual-scale ME features in the embedding space.
- Our Dual-ATME method is extensively evaluated on multiple ME datasets. The experimental results showed that our method demonstrates superior, or comparable, MER performance to state-of-the-art (SOTA) methods on the composite dataset benchmark and single dataset evaluation.

The rest of this paper is organized as follows. Section 2 introduces related work on MER. Section 3 presents the details of our proposed algorithm. Section 4 reports the experimental results on the composite dataset benchmark and single dataset evaluation, as well as ablation studies and visual analysis of our proposed modules. Finally, Section 5 discusses the conclusions and future research directions.

## 2. Related Work

### 2.1. Micro-Expression Recognition

MER is a challenging task that involves recognizing and interpreting subtle facial movements that convey emotions. Generally, these methods could be roughly categorized into hand-crafted methods and deep learning methods.

#### 2.1.1. Hand-Crafted Methods

Hand-crafted methods capture the distinctive features of MEs by manually designing visual descriptors, which are then fed into a classifier for emotion recognition. Local binary pattern from three orthogonal planes (LBP-TOP) [13] combines the temporal and spatial properties of the three orthogonal planes and is broadly deployed in ME analysis. Due to its low computational complexity, many improved methods based on LBP-TOP have been proposed. Liong et al. [14] extracted LBP-TOP based on facial regions of interest (ROIs), which further improved the performance of LBP-TOP. Huang et al. successively proposed Spatiotemporal local quantized pattern (STCLQP) [15] and discriminative spatiotemporal LBP, with revisited integral projection (DiSTLBP-RIP) [16], revealing discriminative information about MEs. Furthermore, using optical flow algorithms to extract motion features for MER has also made adequate progress. Liu et al. [17] proposed using the Main direction mean optical flow (MDMO) to reduce the dimensionality of features. Liong et al. [18] proposed the Bi-weighted oriented optical flow (Bi-WOOF), which applies two schemes to locally and globally weight the Histogram of oriented optical flow (HOOF) [26] descriptors. Bi-WOOF improves the efficiency of MER by representing the motion changes of the whole ME clip using only the disparities of the onset and apex frames. However, hand-crafted feature extraction methods rely on specialized knowledge and complex parameter-tuning processes. In addition, these methods have inferior robustness and find it difficult to adapt to the impact of data change.

#### 2.1.2. Deep Learning Methods

Deep learning-based methods use neural networks to learn and extract features from the data automatically. In recent decades, deep neural networks have achieved prominent results in computer vision and have greatly inspired the exploration of deep learning for MER. The milestone work based on deep learning for MER was [27], which employed CNN networks and transfer learning methods for MER. However, due to the small sample size, end-to-end methods have difficulty extracting salient features directly and their performance did not surpass some hand-crafted methods. However, by using deep learning techniques to further mine hand-crafted features, such as using optical flow as the input of deep network for feature learning [21,22,23,28,29,30,31], the performance of MER further improved. Gan et al. [29] proposed OFF-apexNet, which extracts optical flow features from each video’s onset and apex frames and then feeds the horizontal and vertical components of optical flows into a dual-stream CNN. Based on OFF-apexNet, Liong et al. [19] proposed the Shallow triple stream three-dimensional CNN (STSTNet). Besides the horizontal and vertical optical flows, STSTNet also extracted a hand-crafted feature, i.e., optical strain, achieving the SOTA performance for the composite dataset evaluation benchmark. Zhou et al. [20] designed the Dual-inception Network using horizontal and vertical optical flow features extracted from the onset and apex frames. In FR [32], Zhou et al. fused expression-shared and expression-specific features for MER, further improving performance. In addition, Xia et al. [22,31] modeled the spatio-temporal variation of ME using recurrent convolutional networks (RCNs).

These methods have achieved SOTA performance on MER tasks. In particular, methods based on ROIs improve the performance by selecting the local regions related to ME [33]. However, the small sample size and subtle characteristics of ME samples limit the combination of deep learning and MER. Hence, learning ME features effectively is critical to further improving the performance of MER.

### 2.2. Attention Mechanism in Computer Vision

The attention mechanism plays an essential role in human perception analysis [34,35]. It is based on the principle of assigning higher weights to salient regions in an image and suppressing the useless features. In computer vision, attention mechanisms have gained significant attention because of the ability to effectively focus on the representations of ROIs in images or videos. Recently, these methods have been applied to various visual tasks, such as image classification, object detection and facial expression analysis [36,37,38]. Rodriguez et al. [39] fused spatial attention maps with multi-scale information from diverse convolutional layers to represent the importance of each region in the image. Wang et al. [40] proposed a Residual attention network (RAN) with stacked attention modules to produce attention-aware features. By refining the feature map, RAN achieves prominent performance, while being robust to noisy inputs. Hu et al. [41] proposed the Squeeze-and-Excitation network, using global average pooling features to compute inter-channel attention. Based on this, CBAM [42] exploited both spatial and channel attention modules, further improving the performance of image classification and object detection. CBAM demonstrates that exploiting both spatial and channel attention is superior to using only the channel counterpart.

Furthermore, for MER, attention mechanisms direct the model to focus on critical facial regions and suppress irrelevant facial regions and backgrounds. ME-PLAN [38] combines a 3D residual prototype network and a local attention module to learn a precise prototype of ME features while focusing on local facial movements. Su et al. [43] accentuated the key facial components most relevant to MEs to enhance spatial encoding and capture motive information and non-rigid deformation more effectively. In MMNet [44], Li et al. introduced a Continuous Attention module and a Position Calibration module based on the vision transformer [45], allowing for efficient capture of ME features by focusing on local, subtle muscle movements. Recent methods have also focused on incorporating spatio-temporal and channel information to represent MEs better [46,47,48]. Wang et al. [48] proposed a dual-stream spatio-temporal attention network (DSTAN) that captures MEs’ appearance features and discriminative motion areas. Moreover, DSTAN utilizes a temporal attention mechanism to model the importance of ME clips in a temporal sequence, further boosting the performance of MER. The attention mechanism-based approach described above showed promising results on visual tasks by adaptively enforcing the model to weight different features for the input.

Our attention module draws on the design structure of CBAM, focusing on salient features of facial regions from both channel and spatial perspectives. With this simple and effective design, we improve the network’s discriminative ability for different emotional categories of ME features while avoiding overfitting.

## 3. Proposed Method

### 3.1. Framework Overview

**Figure 1:**
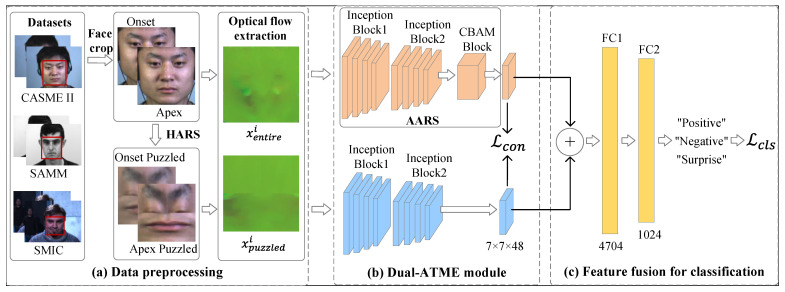
The overview of our proposed Dual-ATME.

Figure 1 illustrates the overview of our proposed Dual-ATME framework, which consists of three modules: Data preprocessing, Dual-ATME module and Feature fusion for classification module. The Dual-ATME utilizes a dual-branch Inception network as the backbone. It employs Hand-crafted Attention Region Selection (HARS) and Automated Attention Region Selection (AARS) to focus on facial discriminative features in manual and automated manners, respectively.

In particular, during data preprocessing, for each original ME clip, we obtain the optical flow features $x_{entire}^{i}$ for the full face and the puzzled counterpart $x_{puzzled}^{i}$ after HARS. Next, in the Dual-ATME module, we feed $x_{entire}^{i}$ and $x_{puzzled}^{i}$ into the dual-branch backbone network for independent feature learning. Then, we execute the contrastive loss $L_{con}$ to pull the distance between HARS-based and AARS-based features from the same ME samples closer, while pushing away the distance between different ME samples. Finally, feature fusion for the ME classification module is performed for expression-refined feature fusion and label prediction.

### 3.2. Data Preprocessing

In this subsection, we introduce the processes of face cropping, HARS and optical flow-based feature extraction.

#### 3.2.1. Face Cropping

In the ME clip, the ME movement starts at the onset frame and reaches its highest intensity at the apex frame. Studies have shown that by comparing these two frames, ME features can be effectively extracted while avoiding redundant information [20,22,49]. Therefore, we only used the onset and apex frames to capture ME movement changes and to simplify the process. In other words, a pair of onset and apex represented one ME sample in our study.

**Figure 2:**
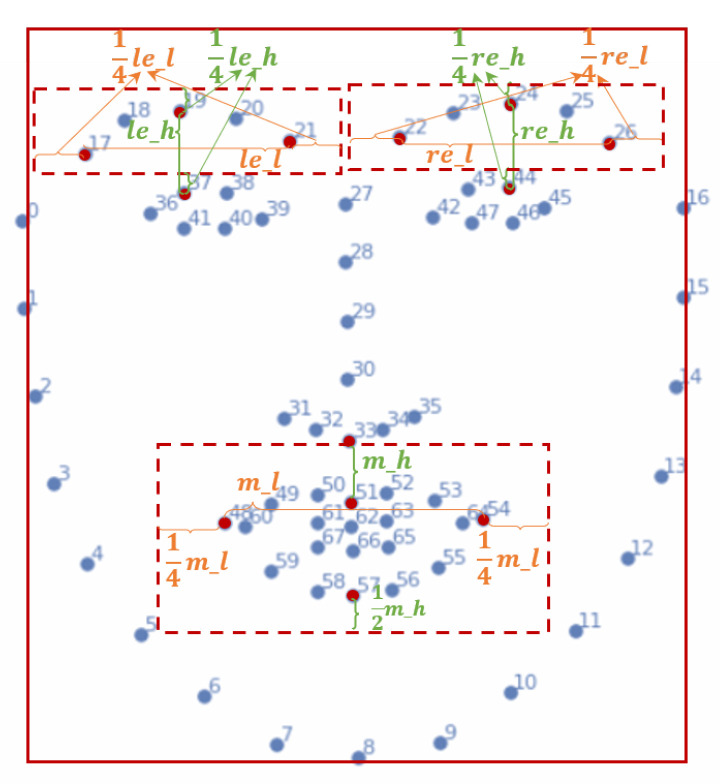
Face cropping and HARS based on 68 detected facial landmarks. Specifically, the eyebrows and the mouth regions were selected for further ME local feature learning.

For each pair of onset and apex frames, we first used the Dlib tool [50] to detect 68 facial landmarks for the onset frame, denoted as $\phi =\{\phi_{i}^{j}|i=1,...,N;j=1,...,68\}$, where *N* represents the total number of ME samples and the range of *j* represents the 68 facial landmarks, as shown in Figure 2.

Then, based on Equation (1), we performed face alignment:

(1) $$\begin{array}{cc} \theta & =atan2(dy,dx)\times 180/\pi \\ \; & =atan2(\phi_{i}^{45}\left(y\right)-\phi_{i}^{36}\left(y\right),\phi_{i}^{45}\left(x\right)-\phi_{i}^{36}\left(x\right))\times 180/\pi \end{array}$$

where atan2() is the azimuth calculation function, dy and dx denote the vertical and horizontal offsets of the line between the two eyes, respectively. We then flipped the face image in the opposite direction of θ and, then, re-performed the face key point detection.

Next, we determined the cropping face region based on the updated coordinates of the facial landmarks. Specifically, for the left, right and bottom boundaries of the face region in *i*th ME samples, we used the landmarks on the sides of the cheeks and the lower jaw, that is, $\phi_{i}^{1}$, $\phi_{i}^{15}$ and $\phi_{i}^{8}$. In addition, for the top boundary, we first calculated the average distance between the left and right sides below the eyebrows and above the eyes, i.e.,

(2) $$d=[\left(\phi_{i}^{38}\left(y\right)-\phi_{i}^{20}\left(y\right)\right)+\left(\phi_{i}^{43}\left(y\right)-\phi_{i}^{23}\left(y\right)\right)]/2$$

Then, the top boundary was defined as $\phi_{i}^{19}+d$, conserving an expansion area above the eyebrows. Finally, we cropped the face region based on these landmarks.

#### 3.2.2. Hand-Crafted Attention Region Selection (HARS)

**Figure 3:**
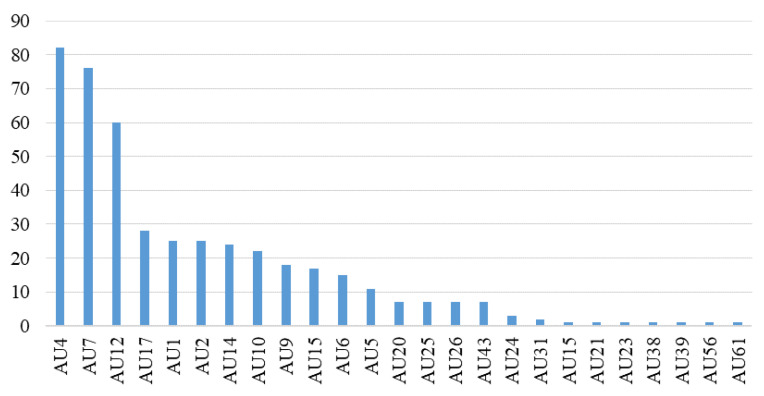
AU statistics for the ME sample of the MECG2019 dataset.

The facial regions with the most muscle activity when MEs occur are the eyebrow and mouth regions [31,51,52]. In other words, these regions contribute most of the discriminative ME information. Furthermore, we conducted a statistical analysis of facial action units (AUs) on ME samples from the CASME II and SAMM datasets of MEGC2019. Based on the AU annotations provided for each ME sample by CASME II and SAMM, we counted and ranked the occurrences of AUs. As shown in Figure 3, it was observed that AUs with higher frequencies were concentrated in the areas around the eyebrows and mouth. Therefore, to reduce the influence of interference from irrelevant regions on MER, our proposed HARS treats the eyebrow and mouth regions as ROIs for local feature extraction. In this way, we can extract more semantic ME-related information.

First, based on the landmarks of the mouth and the eyebrows, we performed region detection and cropping to obtain the boundary borders of each ROI (shown as red dotted boxes in Figure 2). Specifically, for the left eyebrow of the *i*-th ME sample, we calculated the distance between $\phi_{i}^{19}$ and $\phi_{i}^{37}$ as *le_h* and the distance between $\phi_{i}^{17}$ and $\phi_{i}^{21}$ as *le_l*. Then, in order to capture a richer set of information around the left eyebrow, we expanded 1/4·le_l to the left and right sides of the left eyebrow, while expanding 1/4·le_h upwards to the top of the left eyebrow. Additionally, to reduce interference from the eye area, we set the lower boundary of the left eyebrow area to $\phi_{i}^{37}\left(y\right)-1/4\cdot le\_h$. We obtained the boundary of the left eyebrow region through these steps, i.e.,

$$\left[\phi_{i}^{19}\left(y\right)-\frac{1}{4}\cdot le\_h:\phi_{i}^{27}\left(y\right)-\frac{1}{4}\cdot le\_h,\phi_{i}^{17}\left(x\right)-\frac{1}{4}\cdot le\_l:\phi_{i}^{21}\left(x\right)+\frac{1}{4}\cdot le\_l\right]$$

Symmetrically, the boundary of the right eyebrow region was

$$\left[\phi_{i}^{24}\left(y\right)-\frac{1}{4}\cdot re\_h:\phi_{i}^{44}\left(y\right)-\frac{1}{4}\cdot re\_h,\phi_{i}^{22}\left(x\right)-\frac{1}{4}\cdot re\_l:\phi_{i}^{26}\left(x\right)+\frac{1}{4}\cdot re\_l\right]$$

For the mouth area, we calculated the distance between the bottom of the nose ($\phi_{i}^{33}$) and the top of the lips ($\phi_{i}^{51}$) as *m_h* and the distance between the two corners of the mouth ($\phi_{i}^{48}$ and $\phi_{i}^{54}$) as *m_l*. Given the four basic landmarks of the lip ($\phi_{i}^{48}$, $\phi_{i}^{51}$, $\phi_{i}^{54}$ and $\phi_{i}^{57}$), we expanded 1/4·m_l horizontally and 1/2·m_h vertically. Finally, the boundary of the mouth region was

$$\left[\phi_{i}^{51}\left(y\right)-\frac{1}{2}\cdot m\_h:\phi_{i}^{57}\left(y\right)+\frac{1}{2}\cdot m\_h,\phi_{i}^{48}\left(x\right)-\frac{1}{4}\cdot m\_l:\phi_{i}^{54}\left(x\right)+\frac{1}{4}\cdot m\_l\right]$$

Second, after obtaining the boundary coordinates of the eyebrow and mouth regions, we cropped three ROIs for each ME sample: the left eyebrow, right eyebrow and mouth region. Finally, we concatenated the resized left and right eyebrows horizontally and then vertically concatenated them with the mouth. The final puzzled counterpart of the ME sample was formed, as shown in the middle part of Figure 1b.

It is worth noting that, as shown in Figure 1, HARS-based and AARS-based feature extraction are parallel in our proposed network. That is, in addition to the ROI-based puzzled counterpart obtained by HARS, we introduce full face-based AARS, described in Section 3.3.2, thus, achieving MER under the similarity comparison of the two kinds of features.

#### 3.2.3. Optical Flow Extraction

As previously mentioned, the action information in ME is very subtle and it is difficult to extract features of RGB ME samples for MER directly. Based on the theory of brightness constancy [53], optical flow is an appropriate feature to represent action information. We used the TV-L1 [54] algorithm to extract the optical flow features.

Specifically, we estimated and extracted the optical flow information for each ME clip between their onset and apex frames. Inspired by Liong et al. [19], we extracted horizontal and vertical optical flows (u,v) and further added optical strain to form the final optical flow image, which enriches the motion variations of MEs. Specifically, optical strain, as a derivative of optical flow, is capable of approximating the intensity of facial deformation and is less affected by factors such as lighting conditions and skin color. It can be defined as:

(3) $$\epsilon =\frac{1}{2}\left[\nabla O_{f}+{\left(\nabla O_{f}\right)}^{T}\right]$$

where $O_{f}={\left[u,v\right]}^{T}$ denotes the optical flow vector, including horizontal and vertical components and ∇ denotes the derivative of $O_{f}$.

In sum, the final constructed optical flow feature in our study was $\left(u,v,\epsilon \right)\in R^{3\times w\times h}$. For the whole face, we estimated the optical flow feature $x_{entire}$ between the onset and apex frames ($I_{\mathrm{onset}}^{e}$, $I_{\mathrm{apex}}^{e}$ ) using the *TV-L*1 algorithm as follows:

(4) $$x_{entire}=\left(u_{e},v_{e},\epsilon_{e}\right)=TV-L1\left(I_{\mathrm{onset}}^{e},I_{\mathrm{apex}}^{e}\right)$$

Similarly, for HARS-based puzzled counterparts of onset and apex frames ($I_{\mathrm{onset}}^{p}$, $I_{\mathrm{apex}}^{p}$), the optical flow feature $x_{puzzled}$ was obtained as:

(5) $$x_{puzzled}=\left(u_{p},v_{p},\epsilon_{p}\right)=TV-L1\left(I_{\mathrm{onset}}^{p},I_{\mathrm{apex}}^{p}\right)$$

### 3.3. Dual-ATME Module

The Dual-ATME consists of two components: the Dual-branch Inception feature extraction module and the ME feature similarity estimation module. The Dual-branch Inception feature extraction module extracts multi-scale features from the ME sample (full face) and its puzzled counterpart. In particular, we apply AARS on full face-based sub-network to learn ME features effectively. Subsequently, the ME feature similarity estimation module estimates the similarity between extracted features and constrains the similarity of the same group of features to be as high as possible.

#### 3.3.1. Backbone: Dual-Branch Inception Feature Extraction Module

Differences in emotion types and individual expressions for ME can lead to diverse distribution of ME movements on the face. Hence, it is challenging to adapt fixed-size convolution kernels in traditional CNNs to each ME sample. Larger kernels are more suitable for extracting global information, while smaller kernels perform better in extracting local information. To learn both local and global ME features, we used the Dual-branch Inception module as the backbone to extract multi-scale features of MEs based on optical flow input.

**Figure 4:**
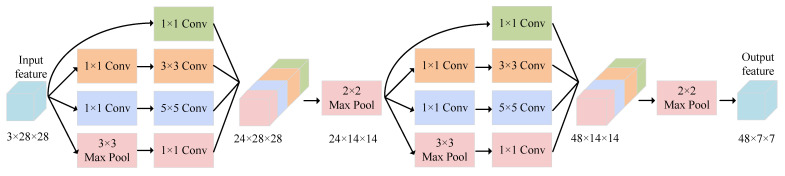
Bi-Inception block in our Dual-ATME.

As shown in Figure 4, each branch of our Dual-branch Inception module consists of a bi-Inception module, composed of two Inception blocks [55]. An Inception block combines feature maps of different sizes and receptive fields, enabling the network to capture richer information. Specifically, the single Inception block uses three different sizes of convolution kernels, namely 1×1, 3×3 and 5×5, to obtain feature maps of various sizes. Additionally, 1×1 convolution layers are inserted between the 3×3 and 5×5 convolution layers to reduce the number of channels in the feature map, thus reducing the computational cost. In our Dual-branch Inception module, the filters for the first and second layers were set to 6 and 12, respectively. Furthermore, we used max-pooling to aggregate the information from the feature maps after each Inception block in each layer. Therefore, in our constructed bi-Inception module, the dimension of the output from the first and the second Inception blocks were 24×14×14 and 48×7×7, respectively.

Following the suggestion in [19], the inputs of the two bi-Inception blocks are optical flow features, including $x_{entire}^{i}\in R^{3\times 28\times 28}$ and its corresponding puzzled counterpart $x_{puzzled}^{i}\in R^{3\times 28\times 28}$, respectively. In particular, as presented in the Dual-ATME module in Figure 1,

- $x_{entire}^{i}$ enters the upper branch, where the bi-Inception network automatically extracts discriminative facial features with the help of the AARS module (See Section 3.3.2).
- $x_{puzzled}^{i}$ enters the lower branch. As mentioned before, the puzzled counterparts are manually obtained with good discriminability through HARS. Moreover, experiments also demonstrated that adding an attention block to this branch did not significantly improve performance (See Table 1). Thus, we did not implement an Attention block in the lower branch to reduce the model parameters.

The core of the Dual-branch Inception module extracts different feature maps by using two deep convolutional layers simultaneously, improving the robustness and classification performance of the model. For the two feature maps extracted by the Dual-branch Inception module, on the one hand, we conducted ME feature comparison learning (described in Section 3.3.3) to pull the ME sample and its puzzled counterpart closer. On the other hand, the two feature maps were cascaded after the Dual-branch Inception module by channel dimension and then sent to the classification module (described in Section 3.4).

#### 3.3.2. Automated Attention Region Selection (AARS)

As introduced in Section 3.2.2, we propose two Attention Region Selection modules, i.e., HARS and AARS. Contrary to manual ROI selection in HARS, AARS exploits the attention mechanism to automatically focus on the crucial features of the original facial region from the channel and spatial dimensions, respectively and assigns higher weights to these features.

Precisely, we deployed the Convolutional Block Attention Module (CBAM) [42] attention mechanism module. CBAM was chosen because it includes not only channel attention but also spatial attention to further enhance valuable features in the feature map and suppresses unnecessary features.

**Figure 5:**
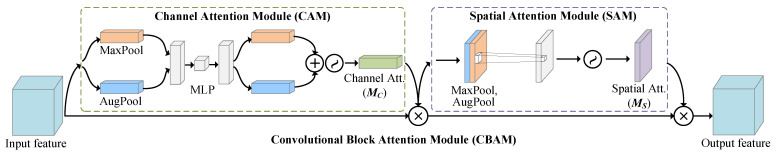
The overview of CBAM. The module has two sub-modules: Channel Attention Module (CAM) and Spatial Attention Module (SAM).

The architecture of CBAM in our study is illustrated in Figure 5. The output of the bi-Inception Module $V_{e\_t}=Inc\left(x_{entire}\right)\in R^{48\times 7\times 7}$ is given as the input to CBAM. We fed $V_{e\_t}$ through the Channel Attention Module (CAM) and the Spatial Attention Module (SAM) in sequence, to obtain the refined features $V_{e}=M\left(V_{e\_t}\right)$.

In detail, CAM outputs the channel attention map using the max-pooling and average-pooling in a weight-shared network, as well as the output of a Multi-layer Perceptron (MLP). SAM pools the output of CAM along the channel axis and passes it through a convolutional layer to obtain the spatial attention map. The CAM and SAM are as follows:

- **CAM**. As shown in the left of Figure 5, we first extracted the spatial context information from $V_{e\_t}$ by passing through the max-pooling and the average-pooling layers. Then, the features were fed into a weight-shared MLP with two hidden layers. Finally, the two features output from the MLP were summed element-wise and the result activated by sigmoid to obtain the channel attention features $M_{c}$. The channel attention weight function $M_{c}\left(\cdot \right)$ can be represented as:
  (6) $$\begin{array}{c} M_{c}\left(V_{e\_t}\right)=\sigma \left(MLP\left(Max(V_{e\_t})\right)+MLP\left(Avg(V_{e\_t})\right)\right) \end{array}$$
  where σ denotes the sigmoid function and Max(·) and Avg(·) denote max-pooling and average-pooling, respectively.
- **SAM**. Subsequently, as shown in the right of Figure 5, the input feature for SAM was $V_{e\_tc}=M_{c}\left(V_{e\_t}\right)\cdot V_{e\_t}$. Then, we applied max-pooling and average-pooling to $V_{e\_tc}$ along the channel dimension. Next, we concatenated the generated feature maps along the channel dimension and applied convolutional and sigmoid operations to obtain the final spatial attention feature map $M_{s}$. The spatial attention weight function $M_{s}\left(\cdot \right)$ can be represented as:
  (7) $$\begin{array}{c} M_{s}\left(V_{e\_tc}\right)=\sigma \left([Max(V_{e\_tc});Avg(V_{e\_tc})\right]) \end{array}$$
  where [·;·] denotes stacking two feature maps along channel dimension.

Finally, the final output feature ($V_{e}$) of the CBAM module (M(·)) can be expressed as:

(8) $$\begin{array}{cc} V_{e} & =M(V_{e\_t})\\ \; & =M_{s}\left(V_{e\_tc}\right)\cdot \left(M_{c}\left(V_{e\_t}\right)\cdot V_{e\_t}\right)\\ \; & =M_{s}\left(M_{c}\left(V_{e\_t}\right)\cdot V_{e\_t}\right)\cdot \left(M_{c}\left(V_{e\_t}\right)\cdot V_{e\_t}\right) \end{array}$$

#### 3.3.3. ME Feature Similarity Estimation

Cosine similarity [56] can be used to measure the similarity or dissimilarity between two embedding vectors and is commonly used in fields such as machine learning, natural language processing and computer vision. In our study, we wanted to measure the similarity between the ME feature maps of the full face and its puzzled counterpart. Hence, we used L2 normalized cosine similarity for the comparison. First, we normalized the two feature maps from −1 to 1. Then, the L2 normalized cosine similarity sim(·,·) was calculated as follows:

(9) $$\begin{array}{c} sim\left(V_{e},V_{p}\right)=\frac{V_{e}\cdot V_{p}}{∥V_{e}∥\cdot ∥V_{p}∥} \end{array}$$

where $V_{e}=M\left(V_{e\_t}\right)=M\left(Inc\left(x_{entire}\right)\right)$ and $V_{p}=Inc\left(x_{puzzled}\right)$. The value of $sim(V_{e},V_{p})$ varies from −1 to 1, where 1 indicates a perfect match and −1 indicates a complete mismatch. If the value is 0, it suggests that the two vectors are orthogonal (perpendicular) to each other and have no correlation. Therefore, to evaluate the difference between two features, we set the contrastive loss as:

(10) $$L_{con}=1-sim\left(V_{e},V_{p}\right)$$

This way, different versions of the same ME sample (full-face and puzzled) were matched in the high-level representation space to achieve instance-level approximation.

### 3.4. Feature Fusion for ME Classification

In the feature fusion stage, two 48×7×7 dimensional features $V_{e}$ and $V_{p}$ from the dual-ATME module are concatenated along the channel dimension to get a 96×7×7 feature. Next, we flatten the feature into a one-dimensional vector and feed it into the final classification module, which consists of two fully-connected layers. To prevent overfitting, we include a Dropout layer with a probability of 0.5 after the first fully-connected layer. The output of the last fully-connected layer is passed through a softmax activation to obtain the ME category predictions.

### 3.5. Joint Loss Function

In the proposed Dual-ATME, the ME feature similarity estimation (contrastive module) and the classification module are jointly trained. Thus, the joint loss function of the entire network is represented as follows:

(11) $$L=L_{cls}+\lambda L_{con}$$

where $L_{cls}$ and $L_{con}$ denote the classification loss and contrastive loss, respectively. λ represents the regularization parameter, which determines the weight of $L_{con}$ in the overall loss. Following the setting in [22], we perform the Focal loss [57] as the classification loss. Focal loss could effectively reduce the loss weight for well-classified examples and focus on complex examples with higher losses, improving the recognition performance on unbalanced datasets. By optimizing the joint loss, Dual-ATME can extract discriminative features for MER.

## 4. Experiments

In this section, we first describe our experimental configuration, which includes datasets, validation protocols and experimental settings. Next, we compare Dual-ATME with SOTA MER methods. We also conducted adequate ablation studies to demonstrate the effectiveness of each module in our framework. Finally, we provide an attention visualization analysis of Our Dual-ATME.

### 4.1. Datasets and Validation Protocols

#### 4.1.1. Datasets

**MEGC2019-CD**. A 3DB-combined dataset called MEGC2019-CD was proposed by Micro-Expression Grand Challenge (MEGC2019) for Composite Dataset Evaluation (CDE). It is a composite of three spontaneous datasets: SMIC, CASME II and SAMM, with three emotion categories: Negative (containing Repression, Anger, Contempt, Disgust, Fear and Sadness), Positive (i.e., Happiness) and Surprise). The detailed information of these three datasets is described as follows and shown in Table 2.

**SMIC**. The Spontaneous Micro-Expression Corpus (SMIC) consists of three different portions captured by different types of cameras: a conventional visual camera (VIS), a near-infrared camera (NIR) and a high-speed camera (HS). We only used the HS subset of SMIC, captured by a high-speed camera and consistent with CASME II and SAMM. The SMIC-HS subset contains 164 video clips from 16 subjects, recorded using a 100 fps high-speed camera with a resolution of 640 × 480. All MEs in SMIC are divided into three categories: Negative (70), Positive (51) and Surprise (43).

**CASME II**. The Chinese Academy of Sciences Micro-Expression II (CASME II) dataset contains 255 ME samples from 26 participants, captured using a 200 fps high-speed camera. The raw resolution of each frame is 640 × 480 and the facial region is 280×340 pixels. The CASME II dataset is divided into seven categories: Disgust (63), Fear (2), Happiness (32), Repression (27), Sadness (7), Surprise (25) and Others (99). In MEGC2019-CD, it is re-classified into three classes: Negative (88, including Disgust and Repression), Positive (32) and Surprise (25).

**SAMM**. The Spontaneous Actions and Micro-Movement (SAMM) dataset consists of 159 ME clips from 29 participants, captured using a high-speed camera at 200 fps. The original resolution for each ME frame in SAMM is 2040×1088 and the facial area is approximately 400×400 pixels. This dataset includes eight raw emotion classes: Anger (57), Contempt (12), Disgust (9), Fear (8), Happiness (26), Sadness (6), Surprise (15) and Others (26). In MEGC2019-CD, it is re-grouped into three categories: Negative (92, including Anger, Contempt, Disgust, Fear and Sadness), Positive (26) and Surprise (15).

#### 4.1.2. Validation Protocols

**Validation Protocol**: Leave-one-subject-out (LOSO) cross-validation is a type of cross-validation where each subject in a dataset is used as the test set once, while the remaining subjects are used as the training set. This method is useful when the subjects in the datasets may have inherent differences. Considering the small sample size in MEGC2019-CD and the significant variation in subjects, we used the LOSO cross-validation method to evaluate the model’s performance.

**Evaluation Metrics**: To evaluate the performance of different methods on the CDE benchmark, we used three metrics: accuracy (Acc), unweighted average recall (UAR) and unweighted F1-score (UF1). These metrics were used to measure the performance on both the composite and individual datasets. The Acc, UAR and UF1 are defined as follows:

(12) $$\mathrm{Acc}=\frac{TP_{c}}{N_{total}}$$

(13) $$\begin{array}{c} \mathrm{UAR}=\frac{1}{C}\sum_{i=1}^{C}\frac{TP_{c}}{N_{c}} \end{array}$$

(14) $$\begin{array}{c} \mathrm{UF}1=\frac{1}{C}\sum_{i=1}^{C}\frac{2\times TP_{c}}{2\times TP_{c}+FP_{c}+FN_{c}} \end{array}$$

where C is the number of classes, $N_{c}$ is the number of samples with the *c*-th class, $N_{total}$ is the total samples and $TP_{c}$, $FP_{c}$ and $FN_{c}$ are the number of true positive, false positive and false negative samples in the *c*-th class, respectively.

### 4.2. Experimental Setting

For each dataset, we used the Dlib [50] tool to detect 68 facial landmarks and used these landmarks to crop the facial regions. During training, the facial images were resized to 28×28 to serve as the input to the network, with random horizontal flipping applied for data augmentation. During testing, the input images were only resized to 28×28 and then fed into the trained model. Our Dual-ATME method was implemented using the Pytorch toolbox, with the backbone network being bi-Inception blocks [55].

The number of training epochs in our Dual-ATME framework was set as 60. The Adam optimizer was employed, with $\beta_{1}$ and $\beta_{2}$ set to 0.5 and 0.999, respectively. The initial learning rate was 0.001 and a cosine learning rate schedule was applied. For each training iteration, 128 ME samples were used in a mini-batch. All experiments were conducted on a single NVIDIA-RTX-4090 GPU. Our code is available at https://github.com/HaoliangZhou/Dual-ATME (accessible since 27 February 2023).

### 4.3. Experimental Results

We compared our proposed method with hand-crafted feature extraction methods and classical deep learning methods on the widely used ME datasets SMIC-HS, CASME II and SAMM. We also performed CDE on their combined dataset MEGC2019-CD.

In terms of the choice of comparison methods, among the hand-crafted feature-based methods, we compared our proposed method with LBP-TOP [13] and Bi-WOOF [18]. Among the deep learning methods, we chose the SOTA methods from the MEGC2019 and MER projects with open source code, including STSTNet [19], Dual-Inception [20], RCN(_a,_w,_c and _f) [22], KFC-MER [43] and MMNet [44]. Given the transient and subtle nature of ME samples, preprocessing operations on the images can significantly affect the results. To ensure comparability and fairness, we reproduced all these methods using the same inputs and data configuration, including the same number of samples, classes and cross-validation protocol. In addition, we used full-face images as the model input when implementing these methods.

Table 3 provides a comprehensive experimental results overview of all methods on the MEGC2019-CDE dataset. Among the hand-crafted MER methods, the best algorithm was LBP-TOP, which had a UAR and UF1 of 0.5753 and 0.5857, respectively. The RCN_a method achieved the highest UAR and UF1 performance of 0.6351 and 0.6339 among the deep learning methods. Besides these methods, our proposed Dual-ATME, combining HARS and AARS, achieved the best performance on the MEGC2019-CD dataset. In addition, it performed the best on all datasets, except CASME II (n.b. shown in bold) and was second best on CASME II, slightly behind RCN_a. In most cases, our method outperformed its competitors significantly, with a UAR and UF1 that were 4.42% and 4.49% higher than the second-best method (RCN_a), respectively.

**Figure 6:**
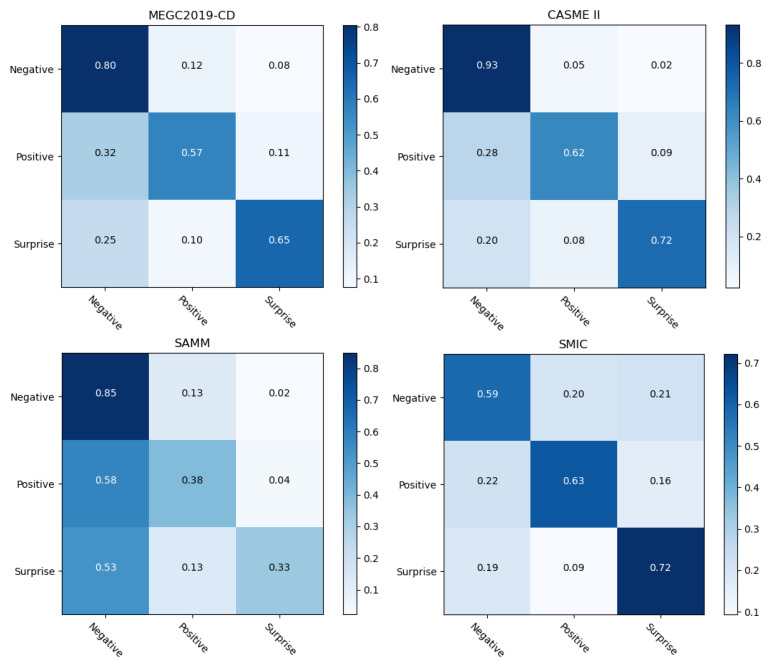
Confusion matrices for MER with Dual-ATME on CASME II, SAMM, SMIC and the MEGC2019-CD datasets.

In addition, Figure 6 shows the confusion matrix of our Dual-ATME on CASME II, SAMM, SMIC and the MEGC2019-CD datasets. On the MEGC2019-CD datasets, Dual-ATME obtained accuracies of 0.80, 0.57 and 0.65 for “Negative”, “Positive” and “Surprise”, respectively. In addition, by comparing the three single datasets, we found that Dual-ATME achieved the highest performance in CASME II, i.e., obtaining 0.93, 0.62 and 0.72 for “Negative”, “Positive” and “Surprise”, respectively. However, in SAMM, Dual-ATME showed the lowest accuracy values in recognizing positive and surprise emotion categories. The reason was that the SAMM dataset had fewer samples than the other two datasets. Moreover, SAMM contains 13 ethnic groups. The rich diversity of subjects, i.e., differences in subjects, can affect the experimental results of LOSO-cross-validation.

From the perspective of different emotional categories, the ME recognition performance on specific emotion type would be empirically improved with more sample data. This is because the deep learning-based model is data-driven, meaning that representational features need to be learned from as much data as possible. As shown in Figure 6, the number of “Negative” samples outnumbered the “Positive” and “Surprise” in each dataset. Thus, the model consistently achieved the highest recognition performance in the “Negative” category compared to the other two categories.

### 4.4. Ablation Study

To demonstrate the effectiveness of our method, we conducted ablation studies to assess the contribution of the proposed modules and components to the final performance. For all experiments, we evaluated the performance using the MEGC2019-CD dataset.

#### 4.4.1. Effectiveness of the Proposed Modules

To evaluate the performance of the crucial modules in Dual-ATME, we conducted ablation studies for HARS and AARS on the MEGC2019-CD dataset. As reported in Table 1, standalone HARS and AARS only achieved sub-optimal performance. Moreover, HARS obtained higher performance than AARS on MER, demonstrating that manual feature extractionm based on prior experiencem plays an essential role in cases of limited data size. The reason is that with limited data, neural networks may not have enough samples to learn complex representations from raw data. In contrast, hand-crafted features, based on prior experience, are more likely to capture the salient information for emotion category identification.

**Figure 7:**
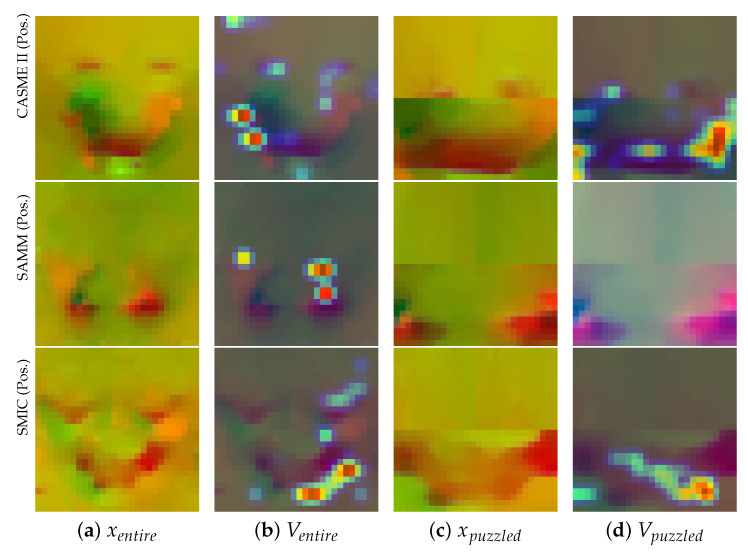
Optical flow (*x*) and Attention map (*V*) visualization in our Dual-ATME of the whole face and puzzled conterpart in CASME II, SAMM, SMIC datasets. (Emotion type: Positive, AU12).

As shown in Figure 7, the Grad-CAM-based [59] feature visualization also proved the above conclusion. Specifically, AU12 represents the upturned corners of the mouth and it was found that the network focused better on the mouth region in the puzzled counterpart. Additionally, the combination of HARS and AARS could achieve collaborative enhancement. As shown in the first row of Figure 7, for this ME sample, AARS only focused on the left corner of the mouth in the full-face image (column (b)). However, the Puzzled image of HARS, based on prior knowledge, was less disturbed, so it not only focused on the left corner of the mouth, but also on the right corner (column (d)). Thus, better MER performance was achieved by fusing the features of HARS and AARS in parallel.

Overall, Dual-ATME combines HARS and AARS and achieved the best results among all variations. HARS-based features are designed to capture concrete, human-understandable concepts, while AARS-based features automatically capture more abstract and data-driven concepts. Combining these two types of features can achieve collaborative enhancement and improve overall performance.

#### 4.4.2. Different Combinations of Face Regions

To validate the effectiveness of the HARS selection strategy based on the eyebrows and mouth regions, we conducted experiments on different combinations of facially discrete areas, including eyebrows, cheeks, nose and mouth. Specifically, we designed the following four combinations: (a) eyebrows + nose, (b) eyebrows + mouth, (c) cheeks + nose, (d) cheeks + mouth and (e) full-face. Thus, the inputs of our Dual-ATME framework were full-face.

As shown in Table 4, the combination of eyebrows and mouth achieved the best performance on MEGC2019CD, SMIC and SAMM datasets, indicating that variations in the eyebrows and mouth regions are more prominent when ME occurs. This result was further supported by the statistical results based on psychological AU annotation shown in Figure 3, which demonstrated the reasonableness and effectiveness of our HARS selection strategy focusing on the eyebrows and mouth regions.

#### 4.4.3. Different Values of Weight Coefficient λ

To evaluate the recognition performance of the proposed method, we varied the value of λ in Equation (11), as listed in Table 5. A larger value of λ meant that the contrastive loss ($L_{con}$) played a more significant proportion of the overall loss and vice versa. Since ME classification is the main task of our model, the classification loss should have the highest weight factor. At the same time, the contrastive loss has an auxiliary role, serving to close the distance between two features in the same group, so we chose a smaller value of λ. In this way, we could balance the effects of both losses and obtain the optimal contrastive performance without sacrificing the classification performance. Specifically, we fixed the weight coefficient of the classification loss to 1 and ranged λ from 0 to 0.1.

The results in Table 5 show that the highest performance was achieved when λ was set to 0.01. It performed the best in all datasets, except for CASME II and SAMM, which still achieved suboptimal competitive performance. This was because if the value of λ is too large, the contrastive loss affects the joint loss function too much, degrading the classification performance. Conversely, a miserly value of λ causes the effect of the contrastive loss to be insignificant, resulting in ineffectiveness in closing the distance between two features.

#### 4.4.4. Different Loss Functions and Optimizers

We also evaluated the performance of different classification loss functions and optimizers. Concretely, we compared the performance of standard CE loss, weighted CE loss and Focal loss, respectively. When deciding the weights of weighted CE loss, we calculated the weights of Negative, Positive and Surprise emotions as 0.2857, 0.1329 and 0.1695, respectively, based on the sample sizes of the three types of MEs in the MEGC2019-CD dataset. We also investigated the improvement of model accuracy by different optimizers, i.e., SGD and Adam.

Table 6 shows the results for Acc, UAR and UF1 using various classification loss functions and optimizers. Focal loss achieved the best performance in MEGC2019-CD, SMIC and SAMM datasets and the competitive sub-optimal performance in the CASME II dataset. It automatically focuses on the categories with the fewest samples and gives these features a higher weight. Meantime, the standard cross-entropy loss achieved the best performance on the CASME II dataset. In our experiments, the Adam optimizer was used by default because it performs better than SDG.

## 5. Conclusions and Perspective

In this paper, we proposed a Dual-branch Attention Network (Dual-ATME) for MER, which consists of three stages: Data preprocessing, Dual-ATME module and Feature fusion for ME classification. In particular, in our Dual-ATME framework, HARS and AARS were combined to extract identity features. Priori experience-based HARS captures noteworthy information about human-understandable concepts. Meantime, Attention mechanism-based AARS automatically captures the complex abstract hidden information within the data. Finally, the above two components were combined to achieve collaborative enhancement and extract discriminative features for MER effectively. The recognition performance of the model was further improved by adding contrast loss in our joint loss to close the two types of features of the same ME sample. Experimental results showed the superiority of our proposed method for performing MER.

In the future, we will further explore automated ROI selection based on large-scale datasets that may help improve MER performance. Specifically, the selection of ROIs is optimized by drawing on the hotspot regions of the attention mechanism. Moreover, we will focus more on spatio-temporal dynamic information to further learn the dynamic representational features of MEs by using the temporal attention mechanism.

## Figures and Tables

**Table 1:** Ablation studies for the proposed modules of our method on the MEGC2019-CD dataset, including HARS and AARS and their combined version, i.e., our Dual-ATME. Note that w/ is an abbreviation for with and A. is an abbreviation for Attention. The best results are highlighted in bold.

Module			MEGC2019-CD		CASME II		SMIC		SAMM	
HARS	HARS (w/ A.)	AARS	UAR	UF1	UAR	UF1	UAR	F1	UAR	UF1
✓	✕	✕	0.645	0.648	0.776	0.785	0.565	0.563	**0.572**	**0.576**
✕	✕	✓	0.633	0.632	0.768	**0.796**	0.533	0.535	0.503	0.516
✕	✓	✓	0.662	0.662	**0.777**	0.784	0.619	0.614	0.515	0.527
✓	✕	✓	**0.680**	**0.679**	0.751	0.765	**0.658**	**0.646**	0.538	0.562

**Table 2:** The emotion categories of MEGC2019-CD.

Datasets	Negative	Positive	Surprise	Total
SMIC [5]	70	51	43	164
CASME II [6]	88	32	25	145
SAMM [7]	92	26	15	133
MEGC19-CD (In total) [10]	250	109	83	442

**Table 3:** Performance comparisons among different methods on the MEGC2019-CD dataset. The best results are highlighted in bold. All the results of the SOTA methods were obtained by reproducing the experiments.

Methods	MEGC2019-CD			CASME II			SMIC			SAMM		
	Acc	UAR	UF1	Acc	UAR	UF1	Acc	UAR	UF1	Acc	UAR	UF1
LBP-TOP [13]	0.643	0.575	0.586	0.786	0.716	0.723	0.555	0.535	0.544	0.594	0.434	0.436
Bi-WOOF [18]	0.661	0.593	0.604	0.773	0.698	0.713	0.592	0.574	0.580	0.624	0.439	0.443
ResNet18 [58]	0.643	0.575	0.586	0.786	0.716	0.723	0.555	0.535	0.544	0.594	0.434	0.436
STSTNet [19]	0.688	0.610	0.624	0.821	0.745	0.769	0.543	0.529	0.532	0.712	0.505	0.531
Dual-Incep [20]	0.680	0.631	0.629	0.814	0.754	0.774	0.575	0.571	0.571	0.649	0.493	0.496
RCN_a [22]	0.681	0.635	0.634	**0.834**	**0.804**	**0.806**	0.567	0.558	0.556	0.654	0.500	0.502
RCN_w [22]	0.661	0.590	0.600	0.758	0.681	0.706	0.567	0.552	0.554	0.669	0.479	0.489
RCN_c [22]	0.681	0.598	0.616	0.779	0.708	0.737	0.573	0.553	0.558	0.706	0.479	0.503
RCN_f [22]	0.667	0.595	0.607	0.772	0.698	0.727	0.561	0.545	0.547	0.684	0.487	0.499
KFC-MER [43]	0.313	0.235	0.255	0.276	0.220	0.229	0.345	0.251	0.283	0.316	0.246	0.240
MMNet [44]	0.601	0.514	0.528	0.766	0.699	0.719	0.457	0.438	0.441	0.594	0.342	0.326
**Dual-ATME**	**0.720**	**0.680**	**0.679**	0.817	0.751	0.765	**0.646**	**0.658**	**0.646**	**0.714**	**0.538**	**0.562**

**Table 4:** Ablation studies for the different combinations of face areas on the MEGC2019-CD dataset. Here, E., F., M., N. are the abbreviations for Eyebrows, Cheeks, Mouth and Nose, respectively. E.&N. is an abbreviation for the combination of Eyebrows and Nose. The best results are highlighted in bold.

Combinations	MEGC2019-CD		CASME II		SMIC		SAMM	
	UAR	UF1	UAR	UF1	UAR	UF1	UAR	UF1
E.&N.	0.630	0.631	0.774	0.781	0.557	0.549	0.487	0.503
E.&M.	**0.680**	**0.679**	0.751	0.765	**0.658**	**0.646**	0.538	**0.562**
F.&N.	0.624	0.631	0.767	0.772	0.527	0.530	0.557	0.566
F.&M.	0.622	0.629	0.760	0.766	0.542	0.545	0.526	0.541
Full-face	0.663	0.670	**0.810**	**0.828**	0.591	0.591	0.543	0.553

**Table 5:** Ablation studies for the different values of λ (represent the balance factor for contrastive loss $L_{con}$) on the MEGC2019-CD dataset. The best results are highlighted in bold.

λ	MEGC2019-CD		CASME II		SMIC		SAMM	
	UAR	UF1	UAR	UF1	UAR	UF1	UAR	UF1
0	0.647	0.646	0.749	0.758	0.602	0.601	0.522	0.518
0.0001	0.668	0.666	0.741	0.755	0.637	0.628	**0.546**	0.559
0.001	0.668	0.668	0.745	0.762	0.635	0.623	0.531	0.547
0.01	**0.680**	**0.679**	0.751	0.765	**0.658**	**0.646**	0.538	**0.562**
0.1	0.649	0.651	**0.788**	**0.794**	0.596	0.594	0.498	0.503

**Table 6:** Ablation studies for the different loss functions and optimizers on the MEGC2019-CD dataset. Note that w-CE denotes the weighted Cross-Entropy loss. The best results are highlighted in bold.

Loss	Optimizer	MEGC2019-CD		CASME II		SMIC		SAMM	
		UAR	UF1	UAR	UF1	UAR	UF1	UAR	UF1
CE	SGD	0.644	0.642	**0.765**	**0.783**	0.591	0.581	0.504	0.516
CE	Adam	0.673	0.671	0.748	0.768	0.643	0.634	0.514	0.527
w-CE	Adam	0.668	0.666	0.751	0.769	0.643	0.634	0.520	0.533
Focal	Adam	**0.680**	**0.679**	0.751	0.765	**0.658**	**0.646**	**0.538**	**0.562**

## Data Availability

Not applicable.
